# Spatiotemporal association between birth outcomes and coke production and steel making facilities in Alabama, USA: a cross-sectional study

**DOI:** 10.1186/1476-069X-13-85

**Published:** 2014-10-23

**Authors:** Travis R Porter, Shia T Kent, Wei Su, Heidi M Beck, Julia M Gohlke

**Affiliations:** Department of Epidemiology, School of Public Health, University of Alabama at Birmingham (UAB), Birmingham, AL 35294 USA; Center for the Study of Community Health, School of Public Health, University of Alabama at Birmingham (UAB), Birmingham, AL 35294 USA; Department of Environmental Health Sciences, School of Public Health, University of Alabama at Birmingham (UAB), Birmingham, AL 35294 USA; Department of Global Community Health and Behavioral Sciences, School of Public Health and Tropical Medicine, Tulane University, New Orleans, LA 70112 USA

**Keywords:** Preterm birth, Low birth weight, Coke production, Steel making, Toxic release inventory, Fugitive emissions, Air pollution, Volatile organic compounds, Polycyclic aromatic hydrocarbons, Metals

## Abstract

**Background:**

Previous research has shown exposure to air pollution increases the risk of adverse birth outcomes, although the effects of residential proximity to significant industrial point sources are less defined. The objective of the current study was to determine whether yearly reported releases from major industrial point sources are associated with adverse birth outcomes.

**Methods:**

Maternal residence from geocoded Alabama birth records between 1991 and 2010 were used to calculate distances from coke and steel production industries reporting emissions to the U.S. Environmental Protection Agency. Logistic regression models were built to determine associations between distance or yearly fugitive emissions (volatile organic compounds, polycyclic aromatic compounds, and metals) from reporting facilities and preterm birth or low birth weight, adjusting for covariates including maternal age, race, payment method, education level, year and parity.

**Results:**

A small but significant association between preterm birth and residential proximity (≤5.0 km) to coke and steel production facilities remained after adjustment for covariates (OR 1.05 95% CI: 1.01,1.09). Above average emissions from these facilities of volatile organic compounds during the year of birth were associated with low birth weight (OR 1.17 95% CI: 1.06, 1.29), whereas metals emissions were associated with preterm birth (OR 1.07 95% CI: 1.01, 1.14).

**Conclusions:**

The present investigation suggests fugitive emissions from industrial point sources may increase the risk of adverse birth outcomes in surrounding neighborhoods. Further research teasing apart the relationship between exposure to emissions and area-level deprivation in neighborhoods surrounding industrial facilities and their combined effects on birth outcomes is needed.

**Electronic supplementary material:**

The online version of this article (doi:10.1186/1476-069X-13-85) contains supplementary material, which is available to authorized users.

## Background

Preterm birth (<37 weeks of gestation) and low birth weight (<2500 grams) increase the risk of early death, long-term disability, and several chronic health conditions including diabetes and cardiovascular disease [1, 2]. In addition, disorders related to preterm birth (PTB) or low birth weight (LBW) accounted for 46% of infant deaths in the United States in 2008 [3]. Previous research examined urban/rural differences in PTB and LBW rates at the zip code level in Alabama [4]. Increased rates of PTB and LBW were found in both highly rural and very urban areas, and these patterns are associated with a high percentage of poverty and a high percentage of individuals identifying as African American or Black [4]. Moreover, significant effect modification was present such that zip code level poverty was more strongly associated with adverse birth outcomes in urban areas (OR 1.23 95% CI: 1.22, 1.24) versus rural areas (OR 1.18 95% CI: 1.14, 1.21), suggesting there may be important environmental factors in impoverished urban areas that increase risk for adverse birth outcomes [4].

Previous research has associated PTB and altered fetal growth with increased exposure to air pollution and developmental toxicity of various components of air pollution have been identified in animal model research [5–8]. Epidemiological studies have revealed difficulties in determining the specific constituents and sources of air pollution that are responsible for adverse outcomes. In studies conducted in the Northeast U.S., LBW in term infants has been associated with the elemental carbon fraction and metal constituents of fine particulates (PM 2.5) [9, 10]. The magnitude of the association was greater for infants with African American mothers in one study [9], but greater in infants with white mothers in another study [10].

Coke and steel production facility air emissions include a mixture of polycyclic aromatic hydrocarbons (PAHs), volatile organic compounds including benzene, toluene, ethylbenzene and xylenes (BTEX), and metals, all of which have been independently associated with adverse birth outcomes in previous research [11–15]. To our knowledge, adverse birth outcomes in relation to coke emissions have not been evaluated in previous literature although coke oven emissions are classified as a known human carcinogen [16] and previous studies have associated occupational exposure to coke oven emissions with increased cancer incidence, decreased lung function, and decreased sperm quality [17]. Previous studies reported that there was no association between emergency department admissions for cardiovascular or respiratory disease and proximity to coke works in England [18], whereas general practice consultations for respiratory disorders was increased in areas closest to coke works in England [19].

Spatial epidemiology studies have demonstrated the utility of mapping concentric buffer zones in classifying proximity to sources of environmental pollutants [20]. For example, previous research has developed census tract level maps for adjusted and unadjusted standardized rates and risks of leukemia in areas surrounding oil refineries in Utah and geographical variation of risk of esophageal cancer in relation to zinc cadmium sulfide exposure in the U.K. [21]. The present study reports the associations between proximity of a mothers’ residence to coke and steel production facilities, yearly emissions from these facilities and adverse birth outcomes. Specifically, we evaluate: 1) the association between a mothers’ residential proximity to coke and steel production facilities and PTB, LBW, and term (gestation >37 weeks) LBW and 2) the association between magnitude of yearly PAH, BTEX, and metal (lead, arsenic, cadmium, manganese, and mercury) fugitive emissions reported to the United States Environmental Protection Agency (USEPA) by these facilities and PTB, LBW, and term LBW.

## Methods

Birth records and geocoding: A total of 517,345 birth records between 1991 and 2010 May-Sept were obtained from the Alabama Department of Public Health (ADPH). Birth records from this dataset reported information regarding date of birth, age of mother, birth weight, gestational age, race and ethnicity of mother, education, method of payment for obstetric fees, birth number (parity), as well as mother’s address, including: state, zip code, city, and street information. Latitude and longitude coordinates of current residence was determined through two rounds of geocoding based on street address and city data using ArcGIS 10.0 (ESRI). Records that could not be linked to latitude/longitude coordinates during geocoding were excluded (N = 100,551). Similar to other research regarding birth outcomes, those recorded as having very low birth weight (<200 grams) or short gestation periods (<24 weeks) were also excluded [22, 23]. The protocol was reviewed and approved through the Alabama Department of Public Health and University of Alabama at Birmingham Institutional Review Boards (UAB IRB protocol # X121023002).

Exposure classification: Yearly emissions data was retrieved from the USEPA Toxic Release Inventory (TRI) database for the entire state of Alabama for the period between 1991–2010 [24]. Briefly, the TRI database includes reports on annual facility emissions of known air toxics, and the records describe the amount and means in which each reported compound is released into the surrounding environment [25]. Identification of 19 coke production and steel making facilities in Alabama were based on primary or secondary North American Industry Classification System (NAICS) codes 324199 (“Other Petroleum and Coal Products Manufacturing”) and 331111 (“Iron and Steel Mills”). Since Standard Industrial Classification (SIC) codes preceded the newer NAICS scheme in the early 1990’s, TRI records before 1997 were selected using corresponding SIC codes 3312 and 2999 (linked to modern NAICS codes using the US Census Bureau Industry Classification System concordance database [26]). Year-specific fugitive emissions records were linked to TRI facility data for mapping and analysis of exposure per total fugitive emissions and compound class fugitive release amount. Compound classes of interest included: PAHs (naphthalene, phenanthrene, anthracene, and polycyclic aromatic compounds (includes 25 additional PAHs), BTEX (benzene, toluene, ethylbenzene and xylene), and metals (arsenic, cadmium, cadmium compounds, lead, lead compounds, manganese, manganese compounds, mercury, mercury compounds). These specific chemical classes were analyzed based on previous evidence of developmental and/or reproductive toxicity [11–15]. To match emissions data with the respective year’s birth records, facilities were separated by release year and mapped with birth records (using lat/long data from geocoding) for only that year using ArcMap 10.0. Facility emissions for each class of compounds were divided into either above or below the median emissions reported for any year across all facilities and all addresses within 5 km of a facility were considered an exposed population. Distance to nearest facility was calculated for each set of birth record coordinates and categorized as less than or equal to 2.5 km, less than or equal to 5.0 km, between 2.5 and 5 km, or greater than 5.0 km. These cutoffs were based on previously used distances between address of residence and emitting facilities to set boundaries defining exposed and unexposed populations [18, 19]. Two facilities, located within the same area, did not have any recorded births within 5 km during the one year that they reported to the TRI database, reducing the number of total facilities to 17 in the final analysis.

Statistical analyses: Logistic regression models were developed to determine associations between LBW or PTB and residence proximity to a coke or steel production facility. For the analysis, births were considered low weight if under 2,500 grams and preterm if gestation was shorter than 37 weeks, following World Health Organization conventions. Covariates for logistic regression models included age of mother, race, education, method of payment for hospital services, and parity. Year of birth was also included as a covariate due to its significant effects on birth outcomes demonstrated in previous work [4]. Age of mother was categorized as <18, 18–35, and >35 years. Race and ethnicity data was collapsed into two dichotomous variables: Black (Y/N) and Hispanic (Y/N). We preserved all original payment categories (private insurance, Medicaid, self-pay, and other). Initial regression models evaluated crude relationships between TRI proximity categories (≤2.5 km,>2.5 ≤ 5.0 km, ≤5.0 km and >5.0 km) and PTB or LBW. Bivariate analyses with birth outcome or distance to TRI facility were conducted for all covariates of interest and those that were significantly associated with either were included in adjusted models. A separate analysis evaluated all LBW newborns meeting inclusion criteria previously described and born at gestational age 37 weeks or greater (i.e., not preterm) since gestational age may modify the effect of exposure on LBW. Twelve of the 17 facilities were in close proximity to at least one other facility; therefore, analyses were also conducted on three areas with facility clusters (Birmingham area, Central Jefferson Co., Tuscaloosa) to examine if there was any facility cluster effects. Emissions-based models evaluated adverse birth outcome associations with facility emissions reported during the year of birth. Statistical analyses were undertaken in SAS v9.3 (SAS Institute; Cary, North Carolina) and maps were developed using ArcMap 10.0 (ESRI; Redlands, California).

## Results

### Descriptive analyses

**Figure 1:**
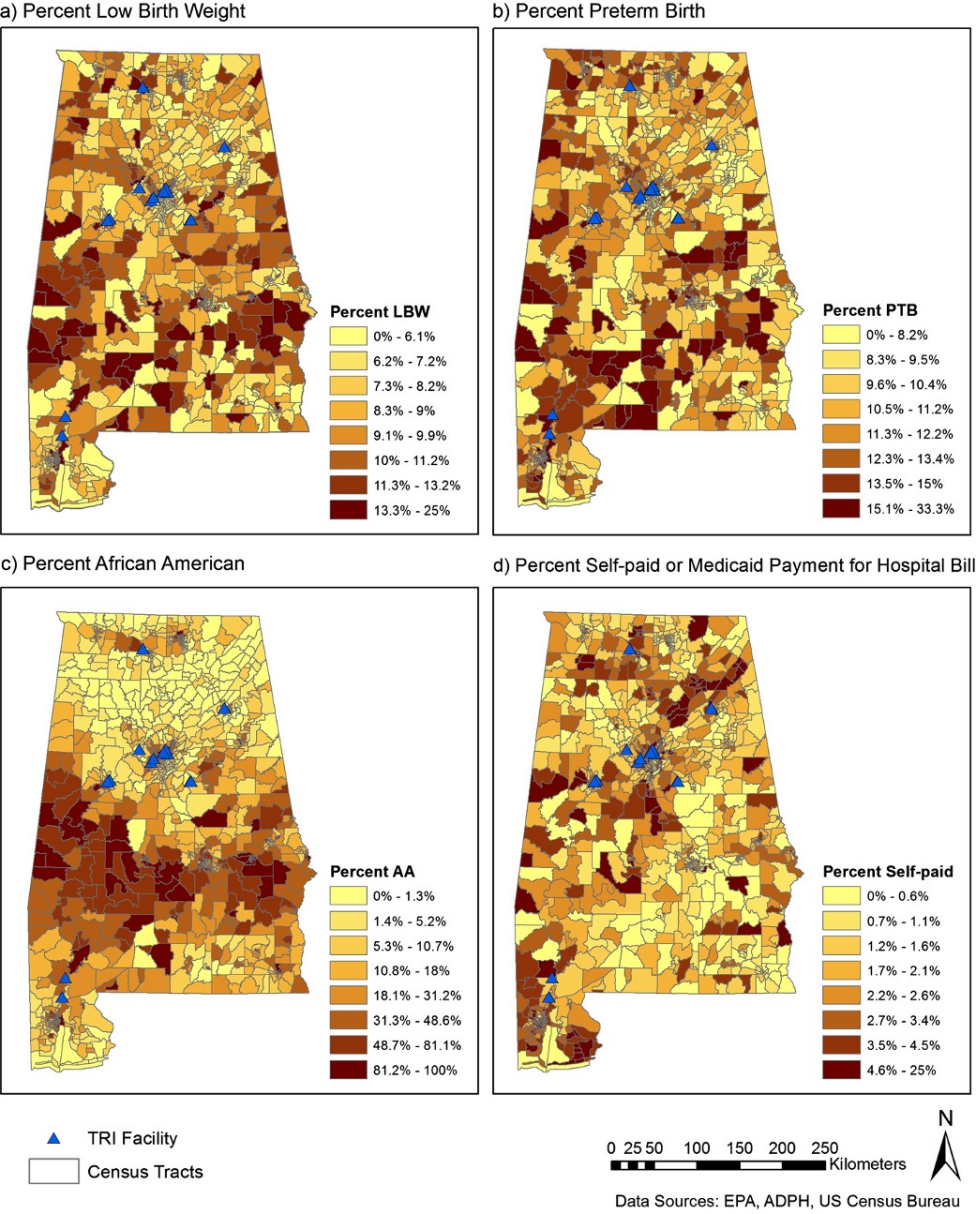
**Census tract level maps of low birthweight (a), preterm birth (b), % African American (c), and % self-paid hospital bill (d) in Alabama, USA.** Locations of coke and steel production facilities with records in the Toxics Release Inventory (TRI) database for at least one year between 1991 and 2010 are identified by black triangles.

**Figure 2:**
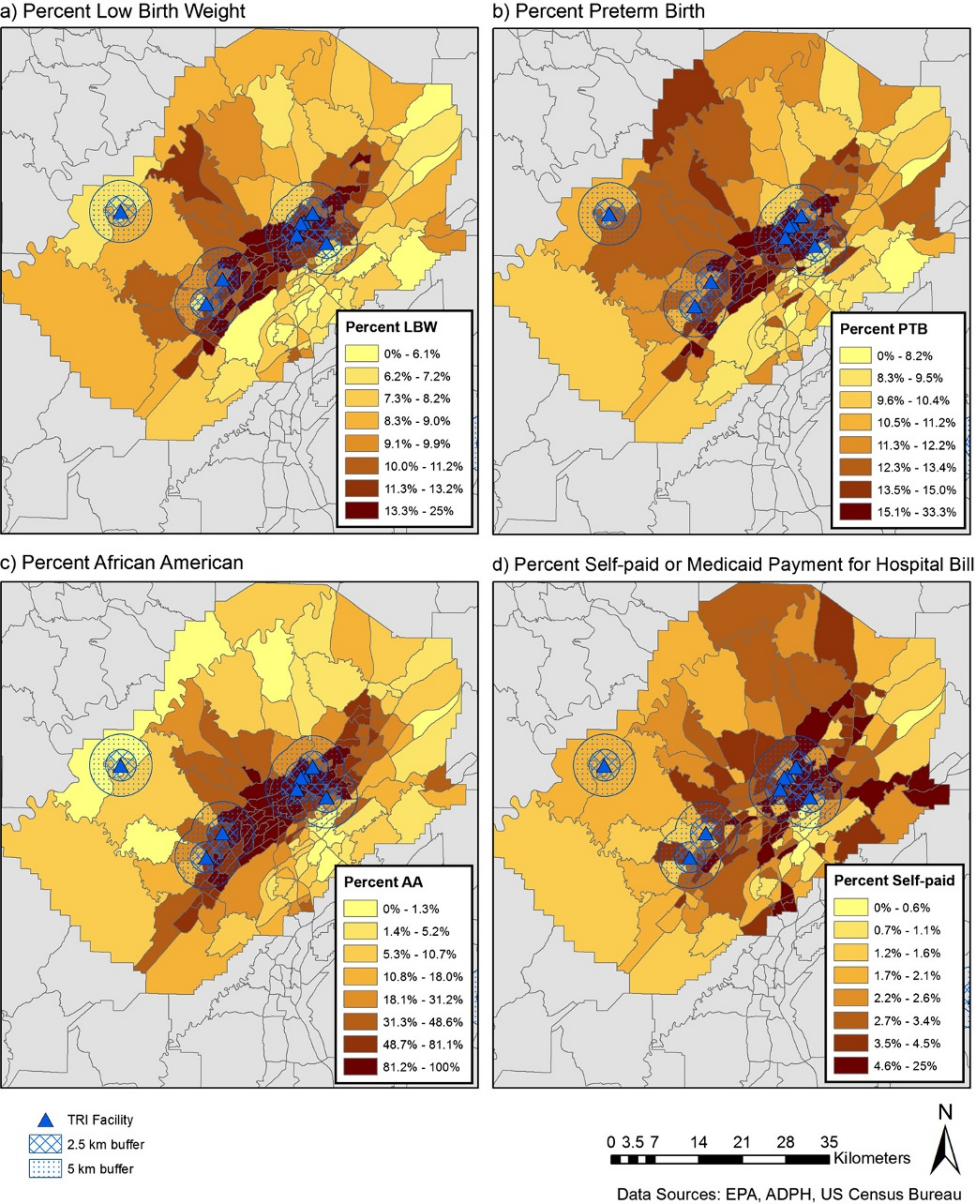
**Census tract level maps of low birth weight (a), preterm birth (b), % African American (c), and % self-paid hospital bill (d) in Jefferson Cty, AL.** Locations of coke and steel production facilities with records in the Toxics Release Inventory (TRI) database for at least one year between 1991 and 2010 are identified by black triangles and concentric circles defining 2.5 and 5 km zones surrounding the facilities are illustrated.

After geocoding and excluding records with missing information, 412,973 records (79.8% of original dataset) were available for analysis (Additional file 1: Table S1). Nineteen percent of records could not be linked to latitude/longitude coordinates during geocoding; therefore characteristics of the final geocoded dataset are compared to the original dataset (Additional file 1: Table S1). A higher percent of more recent records were successfully geocoded. For example, records from 1991 made up 5.1% of the total original dataset and 3.9% of the analyzed dataset; records from 2010 made up 4.8% of the original dataset and 5.4% of the analyzed dataset (Additional file 1: Table S1). In addition, a lower percent of the analyzed versus original records indicated payment through Medicaid or self-pay and education less than 12 years.Overall PTB and LBW rates over the 20 years of data show similar geospatial patterns (Figure 1A and B), with the highest rates in south central regions. Seventeen facilities reporting emissions within Alabama during the period 1991–2010 were identified using the TRI database and locations are plotted in the maps presented in Figure 1. Eight of the 17 reporting facilities are in Jefferson County with 7 clustered within population dense, primarily African American census tracts in Birmingham, AL. Concentric circles defining 2.5 and 5 km buffer zones surrounding the facilities are illustrated in Figure 2.

A higher percent of PTB and LBW occurred to mothers with reported residences within 5 km of coke or steel production facilities when compared to births to mothers with reported residences not within 5 km of these facilities (Table 1). Available covariates (race/ethnicity, payment method, education, and maternal age) also differed by distance from facilities. Additionally, more births in areas within 5 km of the facilities were from the time period of 1991–2000 versus the 2001–2010 period compared to births outside of 5 km, suggesting migration out of areas surrounding facilities or differences in birth rate trends in the two areas (Table 1).

**Table 1:** **Sample characteristics by distance from coke or steel production facility**

	Distance to coke or steel production Facility		
	> 5 km N (col%)	> 2.5 km to 5 km N (col%)	≤ 2.5 km N (col%)
Birth Weight <2500 g			
No	351097 (90.7%)	14252 (88.6%)	8704 (87.9%)
Yes	35854 (9.3%)	1827 (11.4%)	1203 (12.1%)
Gestation Period <37 weeks			
No	342348 (88.5%)	13961 (86.8%)	8533 (86.1%)
Yes	44603 (11.5%)	2118 (13.2%)	1374 (13.9%)
Black			
No	275813 (71.3%)	6539 (40.7%)	3107 (31.4%)
Yes	111138 (28.7%)	9540 (59.3%)	6800 (68.6%)
Hispanic			
No	370914 (95.9%)	15698 (97.6%)	9559 (96.5%)
Yes	16037 (4.1%)	381 (2.4%)	348 (3.5%)
Payment Method			
Medicaid	172019 (44.5%)	8123 (50.5%)	6295 (63.5%)
Private insurance	200862 (51.9%)	7307 (45.4%)	3144 (31.7%)
Self pay	10621 (2.7%)	588 (3.7%)	417 (4.2%)
Other	3449 (0.9%)	61 (0.4%)	51 (0.5%)
Age (years)			
18-35	341824 (88.3%)	13987 (87.0%)	8464 (85.4%)
<18	20078 (5.2%)	1017 (6.3%)	888 (9.0%)
>35	25049 (6.5%)	1075 (6.7%)	555 (5.6%)
Parity			
1	161950 (41.9%)	6892 (42.9%)	4125 (41.6%)
2	132404 (34.2%)	5172 (32.2%)	3089 (31.2%)
3	61666 (15.9%)	2607 (16.2%)	1596 (16.1%)
4 or more	30931 (8.0%)	1408 (8.8%)	1097 (11.1%)
Education (years)			
< 12	82324 (21.3%)	3355 (20.9%)	2867 (28.9%)
12	122443 (31.6%)	5111 (31.8%)	3588 (36.2%)
> 12	182184 (47.1%)	7613 (47.3%)	3452 (34.8%)
Birth Year			
1991-1995	77517 (20.1%)	4784 (29.7%)	3146 (31.9%)
1996-2000	96643 (24.9%)	4321 (26.9%)	2619 (26.5%)
2001-2005	101941 (26.4%)	3449 (21.6%)	2070 (20.9%)
2006-2010	110850 (28.7%)	3525 (22%)	2072 (20.8%)
**Total**	386951	16079	9907

### Inferential analyses

Table 2 presents crude and adjusted odds ratios for PTB and LBW outcomes. A distance-response relationship between TRI facility and PTB and LBW was seen in unadjusted models, but was attenuated after inclusion of covariates. Regression models demonstrated that being an African American mother and payment categories including Medicaid and self-pay (relative to private insurance) was associated with increased odds of PTB and LBW, as well as term LBW (Table 2). Proximity to 1 or more TRI facilities maintained a weak, but significant association with and full term LBW (Table 2). Reported residence of mothers at the time of birth within 2.5-5 km from at least one TRI facility demonstrated a 1.05 (95% CI: 1.00, 1.10) increased odds of PTB and 1.09 (95% CI: 1.00, 1.19) increased odds of term LBW. Living within 5 km of any facility was associated with PTB (OR 1.05, 95% CI: 1.01, 1.09), but not LBW or term LBW. When facilities were analyzed separately or were grouped based on close proximity with each other, living near the six facilities clustered in the Birmingham area was associated with increased odds of PTB (OR 1.07, 95% CI: 1.02,1.13) (Additional file 1: Table S2).

**Table 2:** **Crude and adjusted odds ratios for preterm birth (<37 weeks gestation), low birth weight (<2500 grams) and term low birth weight (<2500 grams and ≥37 weeks gestation) and proximity to coke or steel production facilities in Alabama**

	Preterm birth		Low birth weight		Term low birth weight	
	Crude OR (95% CI)	Adjusted OR (95% CI)	Crude OR (95% CI)	Adjusted OR (95% CI)	Crude OR (95% CI)	Adjusted OR (95% CI)
Distance to nearest TRI facility (km)						
≤ 5.0	1.19 (1.15-1.24)	1.05 (1.01-1.09)	1.29 (1.24-1.35)	1.03 (0.99-1.07)	1.33 (1.24-1.43)	1.04 (0.97-1.12)
> 2.5 - 5.0	1.16 (1.11-1.22)	1.05 (1.00-1.10)	1.26 (1.19-1.32)	1.04 (0.99-1.09)	1.33 (1.22-1.45)	1.09 (1.00-1.19)
≤ 2.5	1.24 (1.17-1.31)	1.05 (0.99-1.11)	1.35 (1.27-1.44)	1.02 (0.95-1.08)	1.33 (1.19-1.48)	0.97 (0.87-1.08)
African American mother						
	1.54 (1.51-1.57)	1.43 (1.40-1.47)	2.04 (2.00-2.09)	1.82 (1.77-1.86)	2.12 (2.04-2.20)	1.79 (1.72-1.87)
Hispanic mother						
	0.73 (0.69-0.77)	0.62 (0.59-0.66)	0.63 (0.59-0.67)	0.56 (0.52-0.59)	0.72 (0.64-0.80)	0.62 (0.55-0.69)
Payment method (compared to private insurance)						
Medicaid	1.28 (1.26-1.31)	1.62 (1.53-1.71)	1.66 (1.63-1.70)	1.24 (1.21-1.27)	2.10 (2.01-2.18)	1.48 (1.41-1.55)
Other	0.92 (0.82-1.02)	1.11 (1.06-1.16)	1.02 (0.90-1.16)	0.98 (0.86-1.11)	1.31 (1.06-1.62)	1.21 (0.98-1.50)
Self Pay	1.64 (1.55-1.72)	1.25 (1.21-1.30)	1.87 (1.77-1.98)	1.81 (1.70-1.92)	1.92 (1.72-2.13)	1.77 (1.58-1.99)
Age (years)						
< 18	1.22 (1.17-1.27)	1.07 (1.05-1.10)	1.44 (1.38-1.50)	1.00 (0.96-1.05)	1.52 (1.41-1.63)	0.82 (0.76-0.89)
> 35	1.25 (1.21-1.30)	0.92 (0.82-1.03)	1.22 (1.18-1.27)	1.41 (1.35-1.47)	1.07 (0.99-1.15)	1.45 (1.34-1.57)
Education (years)						
< 12	1.10 (1.07-1.13)	1.09 (1.06-1.12)	1.19 (1.16-1.22)	1.18 (1.14-1.22)	1.31 (1.25-1.37)	1.31 (1.25-1.38)
≥ 12	0.86 (0.85-0.88)	0.93 (0.90-0.95)	0.75 (0.73-0.77)	0.86 (0.84-0.89)	0.64 (0.61-0.67)	0.78 (0.75-0.82)
Parity	1.14 (1.13-1.15)	1.11 (1.10-1.12)	1.07 (1.06-1.08)	1.02 (1.01-1.03)	0.98 (0.96-1.00)	0.92 (0.90-0.94)
Year	1.02 (1.01-1.02)	1.02 (1.02-1.02)	1.01 (1.01-1.01)	1.02 (1.02-1.02)	1.01 (1.00-1.01)	1.01 (1.01-1.02)

Higher odds ratios for adverse birth outcomes were consistently found for residence proximity to facilities reporting high versus low fugitive emissions of previously identified developmental toxicants during the year of birth (Table 3). High BTEX emissions during the year of birth were associated with LBW (OR 1.17, 95% CI: 1.06, 1.29), whereas high metal emissions were associated with PTB (OR 1.07, 95% CI: 1.01, 1.14), including lead (OR 1.09, 95% CI: 1.03, 1.16) and manganese (OR 1.07, 95% CI: 1.01, 1.14) emissions, also analyzed separately (Table 3).

**Table 3:** Adjusted* odds ratios for preterm birth (<37 weeks gestation) and low birth weight (<2500 grams) associated with living within 5 km of either high or low yearly fugitive emissions from coke or steel production facilities in Alabama between 1991 and 2010

Chemical emissions (lbs. per year)	Low birth weight	Preterm birth
BTEX**		
High (>7,443 – 453,619)	1.17 (1.06-1.29)	1.09 (0.99-1.20)
Low (750–7,443)	0.98 (0.88-1.09)	0.84 (0.75-0.93)
Metals***		
High (>3,256 – 236,459)	1.05 (0.99-1.12)	1.07 (1.01-1.14)
Low (1–3,256)	0.98 (0.92-1.04)	1.02 (0.96-1.08)
PAHs^†^		
High (>1,000 – 81,100)	1.10 (0.98-1.23)	0.99 (0.89-1.11)
Low (1–1,000)	0.96 (0.85-1.09)	0.92 (0.82-1.03)
Lead		
High (>57 – 20,000)	1.03 (0.96-1.10)	1.09 (1.03-1.16)
Low (1–57)	0.98 (0.92-1.05)	1.00 (0.94-1.06)
Manganese		
High (>750 – 13,218)	1.06 (0.99-1.13)	1.07 (1.01-1.14)
Low (5–750)	0.98 (0.92-1.05)	1.04 (0.98-1.10)

*Adjusted for race, ethnicity, payment method, age, education, parity, and birth year.

**Benzene, toluene, ethylbenzene, xylene.

***Arsenic, cadmium, lead, manganese, mercury.

^†^Polycyclic Aromatic Hydrocarbons: Includes emissions values reported for anthracene, phenanthrene, and naphthalene, as well as polycyclic aromatic compounds (includes 25 additional PAHs) in the TRI database.

## Discussion

Previous studies have associated adverse birth outcomes with exposure to air pollution [6, 9, 10, 27, 28] and specific constituents of fine particles [29]. Adverse birth outcomes associated with specific point sources of air pollution have been more difficult to evaluate due to co-location of multiple sources of exposure and low sample sizes [30], although close proximity (<4 km) of residence to incinerators have been associated with increased PTB [31], but not increased odds of all cause and cause specific mortality [32]. Our previous analysis found high poverty and urban zip codes with a high percentage of African Americans had higher odds of adverse birth outcomes even after the addition of individual-level race or socioeconomic factors. These health disparities in birth outcomes are being maintained or increasing over time in Alabama [4]. In the present analysis, higher odds of adverse birth outcomes were seen in neighborhoods within 5 km of coke and steel production facilities after controlling for individual-level covariates available. Maternal residence in neighborhoods surrounding a cluster of 6 facilities in Birmingham, AL was associated with increased odds of PTB. To determine if specific emissions are associated with adverse birth outcomes during that year, associations were determined between adverse birth outcomes and reported fugitive emissions of compound classes that have been previously identified as developmental toxicants. This analysis consistently showed higher yearly emissions were associated with higher odds of adverse birth outcomes and demonstrated that volatile organic compound emissions (BTEX) are associated with higher odds of LBW while high metals emissions, specifically high lead and manganese emissions, are associated with higher odds of PTB.

Limitations of the current analysis include potential residual confounding from unmeasured covariates. We did not have information on infant sex, alcohol, tobacco or other drug exposure, prenatal care, maternal disease status, paternal demographic characteristics, or marital status. Collinearity of distance from facilities with race, maternal age, and socioeconomic factors is present and limits the interpretability of results; however, the consistent results from stratification by fugitive emissions during year of birth provide evidence suggesting exposures to developmental toxicants from coke and steel making facilities may have an independent effect on adverse birth outcomes. The current analysis is also limited by the data availability of warm-season births only and 19% of the records were not able to be geocoded. Relationships investigated here may differ by season since air pollution concentrations and composition vary by season [33] and other industries and traffic-related sources of air pollution are not accounted for in the current analysis. Furthermore, population migration could introduce exposure misclassification [30]. Empirical measurements of exposure would help to further define the relationship between residential proximity to point sources of air pollution and adverse birth outcomes.

Recently, the USEPA has initiated an investigation of the North Birmingham area under Superfund (CERCLA) and Resource Conservation and Recovery Act (RCRA) statutes [34]. Based on the 2010 census, zip code 35207, which encompasses most of North Birmingham where 4 of the 6 Birmingham facilities in the present analysis are co-located, has a population of 11,153. Therefore, estimated risk levels that USEPA uses to determine whether clean-up action is required (1 in 10,000 to 1 in a million) would not be detected in the current study, which is often the case in a small area environmental disease cluster analysis [30].

## Conclusions

Higher odds of LBW and PTB were found in neighborhoods within 5 km of coke and steel production facilities after controlling for individual-level covariates available in the present analysis. Furthermore, a stratified analysis suggests increased odds of adverse birth outcomes associated with high versus low yearly fugitive emissions from these facilities. When analyzed by facility, a cluster of 6 facilities in Birmingham, AL was associated with increased odds of PTB. The present analysis detected suggestive trends justifying further research on the potential compounded burden of social, economic, and environmental stressors, particularly for minority populations.

## Electronic supplementary material

Additional file 1: Table S1: Sample characteristics of original, geocoded, and analyzed datasets. **Table S2.** Adjusted odds ratios for LBW (< 2500 grams), LBW in full term (37 weeks gestation), and PTB births and proximity to specific coke or steel production facilities in Alabama and groups of facilities within close proximity of each other. (PDF 132 KB)

## Abbreviations

PTB: Preterm birth
LBW: Low birth weight
USEPA: United States Environmental Protection Agency
AL: Alabama
BTEX: Benzene, Toluene, Ethylbenzene, Xylene
TRI: Toxic Release Inventory
PAH: Polycyclic aromatic hydrocarbons.
