# Supplementary material for: Spatiotemporal association between birth outcomes and coke production and steel making facilities in Alabama, USA: a cross-sectional study
Source: Environ Health. 2014 Oct 23;13:85. doi: 10.1186/1476-069X-13-85 (PMC4223752; doi:10.1186/1476-069X-13-85)
Supplement: Supplementary file 1 — Additional file 1: Table S1: Sample characteristics of original, geocoded, and analyzed datasets. Table S2. Adjusted odds ratios for LBW (< 2500 grams), LBW in full term (37 weeks gestation), and PTB births and proximity to specific coke or steel production facilities in Alabama and groups of facilities within close proximity of each other. (PDF 132 KB) [file 12940_2014_792_MOESM1_ESM.pdf]

Table S1. Sample characteristics of original, geocoded, and analyzed datasets

	Original Dataset N (%)	Geocoded Dataset N (%)	Analyzed Dataset N (%)
Birth Weight (g)			
< 200	78 (<0.1%)	62 (<0.1%)	N/A
200 - 2499	466,024 (90.1%)	376,093 (90.2%)	374,053 (90.6%)
> 2500	51,030 (9.9%)	40,472 (9.7%)	38,884 (9.4%)
Unknown, Not Stated	213 (<0.1%)	167 (<0.1%)	N/A
Gestational Period (wks)			
< 24	1,777 (0.3%)	1,397 (0.3%)	N/A
24 - 36	454,397 (87.8%)	366,376 (87.9%)	364,842 (88.4%)
> 37	60,247 (11.7%)	48,382 (11.6%)	48,095 (11.7%)
Unknown, Not Stated	924 (0.2%)	639 (0.2%)	N/A
*Distance to nearest TRI Facility (km)**			
> 5	N/A	390,492 (93.7%)	386,951 (93.7%)
> 2.5 - 5.0	N/A	16,287 (3.9%)	16,079 (3.9%)
≤ 2.5	N/A	10,015 (2.4%)	9,907 (2.4%)
Race, Black			
Yes	162,592 (31.4%)	129,111 (31.0%)	127,478 (30.9%)
Unknown, Not Stated	186 (<0.1%)	132 (<0.1%)	N/A
Ethnicity, Hispanic			
Yes	21,573 (4.2%)	17,284 (4.2%)	16,766 (4.1%)
Unknown, Not Stated	277 (0.1%)	212 (0.1%)	N/A
Payment Method			
Medicaid	242,291 (46.8%)	188,152 (45.1%)	186,437 (45.2%)
Private Insurance	253,703 (49.0%)	212,372 (51.0%)	211,313 (51.2%)
Self Pay	15,810 (3.1%)	12,074 (2.9%)	11,626 (2.8%)
Other	4,752 (0.9%)	3,636 (0.9%)	3,561 (0.9%)
Unknown, Not Stated	789 (0.2%)	560 (0.1%)	N/A
Education (years)			
< 12	118,239 (22.9%)	89,469 (21.5%)	88,546 (21.4%)
12	169,997 (32.9%)	132,139 (31.7%)	131,142 (31.8%)
> 12	227,789 (44.0%)	194,261 (46.6%)	193,249 (46.8%)
Unknown, Not Stated	1,320 (0.3%)	925 (0.2%)	N/A
Age (years)			
< 18	29,360 (5.7%)	22,428 (5.4%)	21,983 (5.3%)
18-35	456,466 (88.2%)	367,466 (88.2%)	364,275 (88.2%)
> 35	31,483 (6.1%)	26,891 (6.5%)	26,679 (6.5%)
Unknown, Not Stated	36 (<0.1%)	9 (<0.1%)	N/A
Parity (number of births)			
1	216,345 (41.8%)	174,671 (41.9%)	172,967 (41.9%)
2	175,585 (33.9%)	141,695 (34.0%)	140,665 (34.1%)

3	82,462 (15.9%)	66,476 (16.0%)	65,869 (16.0%)
4 or more	42,823 (8.3%)	33,869 (8.1%)	33,436 (8.1%)
Unknown, Not Stated	130 (<0.1%)	83 (<0.1%)	N/A
Birth year			
1991	26,617 (5.1%)	16,119 (3.9%)	15,986 (3.9%)
1992	25,999 (5.0%)	16,802 (4.0%)	16,652 (4.0%)
1993	26,300 (5.1%)	17,300 (4.2%)	17,090 (4.1%)
1994	25,784 (5.0%)	17,865 (4.3%)	17,672 (4.3%)
1995	25,679 (5.0%)	18,285 (4.4%)	18,047 (4.4%)
1996	25,298 (4.9%)	18,821 (4.5%)	18,622 (4.5%)
1997	25,742 (5.0%)	20,307 (4.9%)	20,090 (4.9%)
1998	26,033 (5.0%)	21,074 (5.1%)	20,855 (5.1%)
1999	26,378 (5.1%)	21,835 (5.2%)	21,590 (5.2%)
2000	26,806 (5.2%)	22,641 (5.4%)	22,426 (5.4%)
2001	25,318 (4.9%)	21,441 (5.1%)	21,286 (5.2%)
2002	24,425 (4.7%)	20,940 (5.0%)	20,780 (5.0%)
2003	24,933 (4.8%)	21,675 (5.2%)	21,465 (5.2%)
2004	24,757 (4.8%)	21,764 (5.2%)	21,573 (5.2%)
2005	25,492 (4.9%)	22,585 (5.4%)	22,356 (5.4%)
2006	26,424 (5.1%)	23,346 (5.6%)	23,120 (5.6%)
2007	27,155 (5.3%)	24,238 (5.8%)	23,923 (5.8%)
2008	26,796 (5.2%)	23,820 (5.7%)	23,713 (5.7%)
2009	26,355 (5.1%)	23,554 (5.7%)	23,453 (5.7%)
2010	25,054 (4.8%)	22,382 (5.4%)	22,238 (5.4%)

*Distance to TRI unknown before geocoding

Table S2. Adjusted* odds ratios for LBW (< 2500 grams), LBW in full term (37 weeks gestation), and PTB births and proximity to specific coke or steel production facilities** in Alabama and groups of facilities within close proximity of each other***.

Facility Grouping	LBW<br>OR (95% CI)	LBW (term)<br>OR (95% CI)	PTB<br>OR (95% CI)
Exposure to Any Facility			
Within 5 km of any facility	1.03 (0.99-1.07)	1.04 (0.97-1.12)	1.05 (1.01-1.09)
*Individual Facility Effects***			
Drummond Co Inc ABC Coke Div	1.05 (0.89-1.23)	1.09 (0.83-1.43)	1.04 (0.90-1.21)
DTE Red Mountain LLC	0.79 (0.49-1.26)		1.03 (0.68-1.57)
Empire Coke Co	1.06 (0.83-1.34)	1.25 (0.85-1.84)	0.76 (0.59-0.97)
Gulf States Steel Inc. of Alabama	1.07 (0.89-1.28)	1.08 (0.80-1.47)	0.90 (0.75-1.08)
Koppers Industries Inc Woodward	1.00 (0.85-1.17)	1.06 (0.81-1.39)	0.93 (0.80-1.09)
Merichem Co	1.46 (0.95-2.22)		1.58 (1.08-2.30)
Nucor Steel Birmingham Inc	1.05 (0.95-1.15)	0.91 (0.76-1.09)	1.16 (1.06-1.28)
Nucor Steel Tuscaloosa Inc	1.12 (0.98-1.28)	1.11 (0.86-1.41)	1.07 (0.95-1.22)
Sloss Industries Birmingham	1.07 (0.67-1.70)	1.31 (0.64-2.68)	1.06 (0.68-1.67)
SMI Steel Inc	0.96 (0.89-1.03)	1.00 (0.88-1.14)	1.03 (0.96-1.10)
USS Fairfield Works	1.06 (0.98-1.15)	1.08 (0.95-1.24)	1.06 (0.99-1.15)
Walter Coke Inc	1.07 (0.87-1.31)	1.00 (0.69-1.43)	1.11 (0.91-1.35)
*Facility Group Effects****			
Birmingham Area	1.00 (0.95-1.05)	0.99 (0.90-1.09)	1.07 (1.02-1.13)
Central Jefferson Co.	1.05 (0.98-1.13)	1.08 (0.95-1.22)	1.04 (0.97-1.11)
Tuscaloosa	1.12 (1.00-1.26)	1.16 (0.95-1.42)	1.02 (0.92-1.14)

*Adjusted for race, ethnicity, payment method, age, education, parity, and birth year

**Facilities with less than 5 observations for any outcome were eliminated

***Facility Groups: Birmingham Area (Drummond Co. Inc. ABC Coke Div., DTE Red Mountain LLC, Nucor Steel Birmingham Inc., Sloss Industries Birmingham, SMI Steel Inc., Walter Coke Inc.); Central Jefferson Co. (Koppers Industries Inc. Woodward Coke Plant, USS Fairfield Works); Tuscaloosa (Empire Coke Co., Merichem Co., Nucor Steel Tuscaloosa Inc.)
